# A Comprehensive Review of Cured Meat Products in the Irish Market: Opportunities for Reformulation and Processing

**DOI:** 10.3390/foods13050746

**Published:** 2024-02-28

**Authors:** Jan Roland G. Molina, Jesús M. Frías-Celayeta, Declan J. Bolton, Cristina Botinestean

**Affiliations:** 1 Food Industry Development Department, Teagasc Food Research Centre, Ashtown, D15 DY05 Dublin, Ireland; 2 School of Food Science and Environmental Health, Technological University Dublin, D07 H6K8 Dublin, Ireland; 3 Environmental Sustainability and Health Institute, Technological University Dublin, D07 H6K8 Dublin, Ireland; 4 Food Safety Department, Teagasc Food Research Centre, Ashtown, D15 DY05 Dublin, Ireland

**Keywords:** cured meat products, Irish processed meats, nitrates, nitrites

## Abstract

Cured meat products constitute one of the meat categories commonly consumed in Ireland and has been part of the Irish cuisine and diet for many years. Ham, gammon, and bacon are some of the products that involve curing as part of the traditional processing methods. Common among these products are high levels of salt and the addition of nitrites. These products undergo processing treatments to create variety, preserve shelf-life, and develop their unique quality and safety characteristics. However, consumers are becoming more conscious of the level of processing involved in these products, and the effects of some components and ingredients might be perceived as unhealthy. Meat product developers have been exploring ways to reduce the amount of ingredients such as salt, saturated fat, and chemical preservatives (e.g., nitrites), which are linked to health concerns. This is a challenging task as these ingredients play an important techno-functional role in the products’ quality, safety, and identity. While innovative processing techniques are being introduced and progress has been made in reformulation and packaging technologies, much is still unknown, especially regarding the applicability of many of the proposed interventions to a wide range of meat products and their sustainability at the industrial scale.

## 1. Introduction

Meat is an important part of the human diet as it is considered an important source of nutrients. Meat is a popular option among consumers as it provides essential nutrients such as protein, amino acids, vitamins, and minerals. The significant increase in global population drives the demand for more available food in the market. In 2021, meat production reached 352 million tons per year globally, which is estimated to be three times the production volume compared to 50 years ago [1]. Continued advancements in the meat industry are shaped by dynamic trends, where consumers have changing needs and expectations from the products they consume. The consumption pattern of meat and meat products among the Irish population is well documented [2,3,4]. According to findings, meat is an important part of the Irish diet. Meat and meat products comprise 17% of Irish adults’ daily energy intake [5].

Irish meat products are renowned for their exceptional quality and sustainable production practices, backed by significant investments from the government. There is also a diverse range of meat products in Ireland that are indigenous and have been traditionally consumed over the years and have also been introduced by global trade. However, with growing consumer demands for healthier and more sustainable products, the meat industry faces challenge associated with high levels of processing; increasing unhealthy appeal for consumers due to salt, fat, chemical additives, and preservatives; and environmental sustainability concerns [6]. Aside from salt and fat, the use of nitrite and nitrate in meat has gained attention recently as they are both considered synthetic chemical additives and are now associated with possible health concerns. There is not yet any available published data on the actual existing nitrite and nitrate levels of cured meat products in the market, but there is an allowable maximum limit of the incorporation of these additives set by the Commission Regulation (EU) 1333/2008 on specific meat products. Though mainly used as preservatives and for other technological functions, it is also recognized that the presence of nitrites and nitrates on food has the risk of the formation of N-nitrosamines, some of which are identified as genotoxic and can induce liver tumors and gastrointestinal cancer in tested animals [7,8]. Based on the latest scientific opinion by an EFSA (European Food Safety Authority) Panel on Contaminants in the Food Chain, meat and meat products are identified as the main food category that contributes to the high exposure of the public to identified carcinogenic nitrosamines, which is a public health concern [8]. The recent legislative changes by the European Union Regulation 2023/2108, which amends the Annex II of Commission Regulations (EU) 1333/2008 and 231/2012 have set significantly lower limits for nitrites and nitrates as food additives to reduce consumer exposure to carcinogenic nitrosamines while still protecting against foodborne pathogens [9]. The recent legislative changes by the European Union Regulation [9] have set significantly lower limits for nitrites and nitrates as food additives to reduce consumer exposure to carcinogenic nitrosamines while still protecting against foodborne pathogens. EU meat business operators have two years to adapt to these new limits. This legislative aspect adds a critical dimension to the discussion, influencing the direction of research and the development of alternative solutions.

Numerous research studies have been conducted with the aim of exploring the reformulation of meat products to improve the nutritional profile of some Irish meat products. This includes studies on the reduction and replacement of ingredients that consumers are becoming wary about, such as salt, saturated fat, chemical additives, and preservatives [10,11,12]. It is a challenge to meat product processors to develop such products as the quality and safety parameters are affected by these reformulations. However, there is still a lack of studies on the safe use of nitrites and nitrites taking into account the Irish production methods and balancing concerns on nitrosamine formation and the microbiological safety of the products [13]. It is also important to consider that the development of products should consider the existing context of the target market. Important stakeholders are the consumers and the industry adopting the technologies. 

This review aims to provide insights on the processing aspects of the cured meat category in the Irish market. It intends to explain the role of processing treatments and chemical additives in this product category and the challenges encountered on the demand to reduce and replace ingredients with essential techno-functional properties in relation to different meat matrices. It also highlights recent advancements in meat product development and identifies opportunities and strategies for reformulation and innovative processing and preservation methods.

## 2. Cured Meat Products in the Irish Market

**Figure 1:**
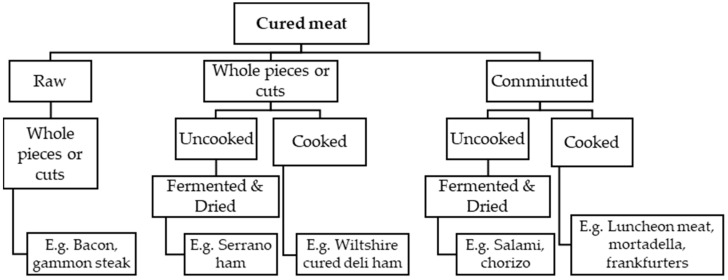
Classification of cured meat products in the retail market.

Cured meat products constitute one of the categories of the larger group of meat products. The term ‘meat products’ refers to “processed products resulting from the processing of meat or from the further processing of such processed products so that the cut surface of the original product shows that the product no longer has the characteristic of the fresh meat [14]”. Commonly, other terms such as ‘processed meat’ or ‘processed meat products’ are also used to pertain to this category. However, it should be noted that there are also other processed meat products that have not necessarily undergone curing. Cured meats are characterized by the inclusion of sodium and/or potassium salts of nitrates and nitrites in the ingredients list. In Ireland, pork comprises most of the cured meat products, and to some extent, beef and some offal are also available as cured product in the retail market. Some of the commercially available meat products are considered and labelled ‘traditional’ or ‘traditional Irish’, such as ham, bacon, and gammon. Bord Bia, Ireland’s state agency on the promotion and marketing of Irish food and horticulture, issues the Quality Mark on Irish products, which assures consumers that the meat has been produced to the highest level of standards in terms of safety, traceability, and farming welfare. The categorization of cured meat products is presented in Figure 1. 

Processing of cured meat starts when the animal carcass is obtained after the slaughtering. The initial steps include removal of the hide, head, feet, and offal of the animal [15]. Traditionally, skilled butchers manually cut the meat from animal carcasses into primal, sub-primal (fabricated cuts), and retail cuts. The structural characteristics of the animal muscle is still intact on these raw whole pieces and cuts. Table 1 shows some of the retail cuts of meat from pork and beef that are typically cured. High level of skills and hygienic practices are needed in order for this to be efficient. Safety, quality, consistency, and productivity are crucial factors in meat preparation, which involves tedious and meticulous tasks [16]. 

Bacon, ham, gammon, and corned beef are common Irish cured meat products that use whole meat pieces. These products are characterized by the primal cut used for curing. For instance, pork side (belly portion) and loin (back part) are used in the production of streaky and back bacon rashers, respectively. The hind legs and sometimes the fore legs of pork are used in ham and gammon. Brisket, which is obtained from the breast or lower chest of the cattle, is cured to make corned beef. Raw cured meat pieces require cooking before consumption. For example, bacon is usually sold raw, vacuum packed, or packaged in modified atmosphere and stored at chilled temperature. Depending on preference, the consumer can choose to either grill (i.e., no additional fat is used), shallow fry, or fry the product. Large cured meat cuts such as gammon steaks, bacon joints, and corned beef are usually roasted or boiled just before consumption. On the other hand, comminuted meat comes from meat pieces or trimmings that have undergone further size reduction by means of mincing into fragments or passing through a spiral-screw mincer. Protein and fat ratios are important parameters that would dictate their value in the market. These products are usually used in sausage-types or emulsion-types of meat products. These are further dried and fermented and/or cooked. The term ‘further processed’ was used in the meat products classification system presented by Seman et al. [18]. This group of products has undergone ‘transformation beyond minimal processing’, such as additional preservation and processing steps to enhance sensory, quality, and safety attributes. Mincing and mixing processes extensively alter the structural characteristics of the meat by size reduction. This leads to a uniform distribution of curing and flavoring agents before the product is restructured to form a meat gel matrix. Pre-cooked cured products such as cooked emulsified meat (e.g., frankfurters) and pudding are re-heated by shallow frying or grilling. Several cured meat products can be readily eaten upon opening the package. For example, cooked deli hams and other deli-style meat slices (e.g., sliced luncheon rolls, sliced corned beef, mortadella slices) and slices of dry-fermented or salted meat products (e.g., sliced salamis, prosciutto, Serrano hams) are usually eaten directly as sandwich fillings. Usually, these types of products are packed in modified atmosphere packages with a mix of inert gases in order to preserve the freshness of the product. Table 2 shows examples of the cured meat products available at the leading retail markets in Ireland.

### 2.1. Curing Ingredients

The ingredients common among all cured meat products are salt (sodium chloride) and curing agents such as nitrates and nitrites. Such curing agents are considered additives in the EU and are assigned with E numbers that are mandatorily declared in the label of the packaged products. The potassium and sodium salts of nitrates and nitrites are food additives allowed for use in cured meat: potassium nitrite (E249), sodium nitrite (E250), sodium nitrate (E251), and potassium nitrate (E252) [19]. Depending on the product, curing adjuncts such as sodium ascorbate or erythorbate and salts of phosphates are also used to aid the curing process and to enhance the texture and shelf-life. For some fermented-cured meat and cure-in-bag products, sugars, in the forms of sucrose and dextrose, are added as substrates of starter cultures or naturally occurring desirable meat microflora for the production of lactic acid and other flavor compounds.

Salt has been used for many years on meat products, mainly for preservation. It is well understood that it works by decreasing the water activity of food and thus controlling the growth of pathogens and spoilage microorganisms. Salt also controls the enzymatic reactions in the ripening and maturing of fermented sausages. In addition, it also contributes to the distinct meat taste. Sodium chloride also plays an important role in curing as it also participates in the reactions involving nitrites and myoglobin, which is the meat pigment [20]. For minced meat products, it helps in the solubilization of myofibrillar proteins, which have an impact on the desired texture in the final product [21]. A survey on the declared salt contents of commercially available bacon and ham in Ireland conducted by Delgado-Pando et al. [22] revealed that the average salt contents of these products are 2.60 g/100 g for bacon and 2.13 g/100 g for ham.

Salts of nitrates and nitrites are labelled as preservatives on meat product packages. Preservatives are used to delay early spoilage and extend the shelf-life of meat products by preventing the growth of undesirable microorganisms. *Listeria monocytogenes* and *Clostridium botulinum* are two of the target pathogens [23]. However, the function of nitrites is not limited to antimicrobial properties; it also enhances other quality parameters of the product, such as color and flavor. Upon addition into meat, nitrite reacts with hydrogen ion to produce nitrous acid that progressively decomposes into water molecules and dinitrogen trioxide, which then generates nitric oxide and nitrogen dioxide [24]. The primary component responsible for the cured pink color in meat products is nitric oxide, which combines with the iron of myoglobin and metmyoglobin to produce the desired color [25]. Nitrite acts as an antioxidant by protecting the lipid molecules of meat from oxidation by several proposed mechanisms. Nitrite can chelate metallic ions, which are the main pro-oxidants in meat, and thus stabilizes the heme iron of the myoglobin. In addition, nitrite is also reactive to reactive oxygen species and prevents initiation of lipid oxidation, while nitric oxide also reacts with lipid peroxyl radicals and produces non-radical molecules to break the oxidation chain-reactions [26]. On the other hand, nitrites are found to have both antioxidant and pro-oxidant effects on proteins. The study by Feng et al. [27] shows that the increasing amount of nitrites results in lower protein carbonyl content, higher free amines, and lower surface hydrophobicity, and at the same time it decreases sulfhydryls and increases disulfide bonds in cooked sausages. In contrast, the addition of nitrites on salted and cured beef resulted in the reduction of disulfide bonds, dityrosine, surface hydrophobicity, and the transformation rate from α-helix to β-sheet protein conformation, but the sulfhydryl group content of myofibrillar proteins was significantly increased [28]. It is generally recognized that nitrites influence the meat flavor, but the exact mechanisms of this is not yet fully understood. It was hypothesized that, due to the protective ability of nitrites towards the oxidation of lipids, the undesired warmed-over flavor is prevented, leading to the perception of other flavor compounds (i.e., Strecker aldehydes and Maillard reaction products) produced from the oxidation of proteins [20]. Nitrate, in its sodium or potassium salt and commonly called *saltpetre*, is not known to be directly involved in the color fixation and flavor development reactions of cured meat products. Nitrates should be converted into nitrites to further undergo the curing reactions in the matrix [25].

Sodium ascorbate (E301) is also used in cured meat products, and it is declared as an antioxidant on the packages. Ascorbate coexist with its acid form and helps facilitate the faster conversion of nitrite into nitric oxide, and also the reduction of ferric ion (Fe^3+^) to ferrous (Fe^2+^) ion [20]. This effectively enhances the attachment of nitric oxide to the meat pigment myoglobin, thereby stabilizing the desired cured color [29]. In this regard, other authors would term sodium ascorbate as a ‘curing accelerator’. The addition of ascorbate in a cured meat model system increased the cured meat pigment content [30]. This is usually mixed in the brine solution, together with other curing ingredients such as salt and phosphates. It is suggested that the preparation of curing brine containing ascorbate and phosphates should be carried out immediately before use to avoid losing nitrites [31]. Results by Villaverde, Morcuende, and Estévez [32] suggests that 500 ppm ascorbate can compensate the pro-oxidant impact of nitrite on meat products. A separate study elucidates the importance of ascorbate to control the protein carbonylation in dry-fermented sausage without declining the formation of Strecker aldehydes. The addition of sodium ascorbate contributed to the lowering of lipid oxidation; however, it increased the protein carbonyl content of dry-fermented sausage [33]. Other alternatives for sodium ascorbate are sodium erythorbate, which is its isomer, and polyphenols.

### 2.2. Processing Aspects of Various Cured Meat Products

#### 2.2.1. Irish Bacon, Ham, and Gammon

Bacon is a cured meat product that uses the middle back part of the pig carcass, which has the loin or back part or the *longissimus* muscles. This is known as ‘back bacon’ or ‘rashers’ in both Ireland and the United Kingdom. Medallion cuts are the leaner variants of bacon because the fat surrounding the loin portion is trimmed, resulting in 50% less fat content compared with the usual back bacon, as claimed on the label. In other variants, the belly portion is used, and this is called ‘streaky bacon’ due to the strips and layers of fat and muscle and is composed of layers of muscles (*cutaneous trunci*, *latissimus dorsi*, *pectoralis profundus*, *rectus abdominis*, and internal and external abdominal oblique) and intermuscular fat [34]. Rashers, medallion, and streaky bacon variants are in the form of thin cuts of approximately 2.3 to 2.8 mm, with 5 to 7 pieces per package. These thin cuts of raw bacon are usually packed in plastic packaging under a modified atmosphere. Bacon chops, on the other hand, are the thicker version of back bacon of approximately 12 to 15 mm. Consumers usually need to grill or pan-fry the bacon, as instructed on the package, before consumption. Bacon joints and bacon eye loins are larger cuts of the loin, of approximately 1.5 to 3 kg, and they are cured in the packaging and are also known as ‘cure-in-bag’ meat products. These are packaged raw in a vacuum bag and the curing agents were already incorporated prior to packaging, and maturing (i.e., allowing the curing to take place) takes place upon storage. All these bacon variants of varying cuts and sizes have smoked and unsmoked variants. Aside from smoking, other flavors, such as paprika and orange blossom, smoked dry-cured whiskey, maple syrup and cinnamon glazed, and orange blossom and honey and ginger, are also incorporated to bacon to create more variety. Lardons are the value-added pieces of meat derived from cooked bacon or dry-aged meat.

Among the cooked meat category, cooked ham is a popular meat product in Ireland. Ham is the general term for the upper leg and buttock of the pig. This is also another Irish traditional cured meat product that uses hamstring muscles such as the semitendinosus, semimembranosus, and biceps femoris of the pig carcass. Usually, the term ham is used when the product is sold as cooked up to 72 °C internal temperature after the curing and maturing process and is packaged as ready-to-eat. This is also known as deli ham as it is usually served in delicatessen shops for sandwiches and salads. Cooked ham is available in more varieties in terms of the thickness of cuts. The terms ‘hand cut’, ‘carved’, ‘large slice’, ‘shaved’, and ‘wafer thin’ are used to describe different cuts of cooked ham. Like bacon, flavorings (e.g., chipotle and barbecue) and flavoring techniques such as smoking, roasting, oven baking, and slow cooking are also applied. Several glazing options such as honey, firecracker (sweet and spicy oriental sauce), honey roast, and orange and wildflower also creates variety. On the other hand, the term gammon is used for the raw whole muscle of ham, in larger portion cuts, that is cured and packaged raw in a vacuum bag. It can also be smoked or unsmoked. Consumers need to cook it by roasting or boiling at a specified time and temperature before consumption. 

##### Traditional Curing of Irish Bacon, Ham, and Gammon—Wilshire Curing Process

Commercial cooked ham, gammon, and bacon labelled as ‘traditional’ are produced using the Wiltshire curing process. This traditional process is given recognition by the European Union in Regulation 1333/2008 under Annex II in the food category 8.3.4.1. The specifications of this traditional process are broadly described as follows: “meat is injected with curing solution followed by immersion curing for 3 to 10 days. The immersion brine solution also includes microbiological starter cultures [19]”. The traditional Wiltshire wet curing process involves injecting 8–10% (*w*/*w*) of the whole muscle of pork with curing solution [35]. In injection curing, the brine is pumped and injected directly into the meat pieces using multiple needles. This reduces the risk of spoilage as less curing time is required, and the meat is also mechanically tenderized by puncturing it. The injected meat is then subsequently immersed fully in ‘live brine’, a brine that contains live salt-tolerant microorganisms [18]. Both the injection curing and the wet immersion curing contain sodium chloride and curing agents such as nitrate (sodium or potassium salt) and sodium nitrite. The use of this microbiologically active immersion curing brine makes it unique among the processes of ham production. The exact profile of the microbial composition of this live brine was not really clear until Woods et al. [36] made a study on the microbiome analysis using next generation sequencing (NGS) technology. It revealed that the identified genera *Marinilactibacillus*, *Carnobacterium*, *Leuconostoc*, and *Vibrio* were the core microflora present in Wiltshire curing brine. These are known to be salt-tolerant microorganisms responsible for the flavor development. Immersion curing takes from 3 days to 10 days, maintaining a temperature of approximately 4–5 °C. The cured meat is then drained and matured for a minimum of five days. Limited studies have been conducted on this aspect of this special type of wet curing. As the live brine contains certain amounts of nitrates and nitrites, the reduction or removal of these chemical curing agents have not been studied in terms of the effect on the microflora and consequently on the overall quality aspects of the finished products. If the refrigerated temperature is not maintained or contaminated curing solution is used, the spoilage of meat (especially near bones) can occur. The solution also becomes ropy during storage, imparting an off-flavor to the product. 

The smoking of ham and bacon meat pieces is a common option to add the desired smoked flavor of the product. This process also reduces moisture to some extent, and smoke constituents provide preservative and antioxidant actions. While concomitantly harmful substances (e.g., PHAs, HAAs) can also be produced, they can be removed by introducing water sprays or electrostatic precipitation between smoke generation and deposition on to the product [37]. Smoke from hardwoods such as oak, beech, and hickory are used [38]. Modern development of the smoking process involves the generation of smoke from shavings or sawdust contained in a separate unit of the cooking chamber. Cold smoking is applied in the range of 35 to 50 °C, while hot smoking is over 80 °C [37]. Smoking is carried out over a range of 4 to 12 h, depending on the desired intensity of the flavor and dryness. After smoking, the product should be rapidly cooled to avoid the undesirable growth of microorganisms [39]. Smoke concentrates (i.e., liquid smoke) are also sometimes used. The results of the study by Yin et al. [40] indicate that sausages treated with industrial liquid smoke show the highest level of phenolic compounds, resulting in a more smoky aroma of the products relative to other products from traditional smouldering and industrial smouldering methods of smoking.

The products produced under this traditional processing method are below the derogated meat products in terms of the nitrate and nitrite content regulation of cured meat products. Under this current regulation, the maximum residual nitrate and nitrite are regulated, but not the input amount in the formulation [41]. This gives flexibility for the manufacturers to regulate the necessary amounts of nitrates and nitrates that would work on their existing formulation and process. Based on the latest change on nitrite and nitrate limits in the EU, the permitted maximum residual nitrite ion level of Wilshire ham is 65 mg per kg, while it is 150 mg per kg for nitrate ion. On the other hand, 105 mg per kg is the permitted maximum residual nitrite ion level, and it is 150 mg per kg for nitrate ion for Wilshire bacon [9].

##### Non-Traditional Curing Processes

For non-traditional production processes, the texture of brine-injected meat is further enhanced by massaging and tumbling. These are mechanical processes that ensure the quick diffusion and binding of curing ingredients and allow a greater pick up and retention of moisture by the meat. The mechanical tenderization of whole meat pieces and cuts also leads to physical destruction of the structural characteristics of the meat fibers; thus, cure ingredients are distributed faster [42]. Extracted proteins bind muscle pieces together due to reduced extracellular spaces, increased degradation of muscle structure, and the emergence of amorphous zones [43]. This function is also utilized in the preparation of restructured cured products. The effect of tumbling and massaging is further enhanced if a vacuum is applied during agitation. 

#### 2.2.2. Cured Beef and Offal—Traditional Corned Beef and Cured Ox Tongue

Corned beef is also a known traditional Irish meat product that is usually paired with cabbage and is consumed during special occasions such as Christmas, Easter, and St. Patrick’s Day [44]. Corned beef is made with Irish beef brisket (i.e., beef lower chest), salt, and nitrate salt [45]. The use of the term ‘corned’ means ‘corns of salt’, which are added to the whole meat piece for preservation. Sodium nitrite is now being used as it is the readily available form of nitrate for preservation and as a color retention agent, together with curing adjuncts such as sodium ascorbate. The brine solution, containing salt and curing agents, is pumped into the raw brisket by injection, and the meat is then cured in a netted bag. Corned beef is usually sold raw and stored at chilled temperature. Consumers cook the product by boiling or simmering for a specified time. The other formats of corned beef are pre-cooked and sliced and are packed in modified atmosphere packaging similar to that in deli hams. Canned (i.e., cooked in a hermetically sealed container) corned beef, which is ready-to-eat, is also available. Canned corned beef is hashed beef exposing the meat fibers and tender cartilages, and it has a longer shelf-life due to the sterilization processed applied. Though available in Ireland and in Europe, canned corned beef is more popular in the North and South American markets.

Ox tongue is also an Irish traditional meat product. The product involves curing with the incorporation of gelatine. The results of the study by Warren, Bowker, and Mohan [46] reveal the potential of ox tongue for improving the protein, fat, and nutrients of meat products. Similar to cooked ham, ox tongue is also sliced thinly and is packed in a modified atmosphere, or is canned. In the European Union, the maximum limit for the residual nitrite (as the ion form) of ox tongue produced under traditional methods (i.e., immersion cured for at least 4 days, and pre-cooked) is 7 mg per kg [9]. 

#### 2.2.3. Dry-Fermented/Salted Meat and Sausages

Comminuted cured and fermented meat products such as salami, pepperoni, and chorizo are considered uncooked or raw, but ready-to-eat. These comminuted cured meat products are mixed with salt, curing agents, spices, and seasonings and are filled into casings before fermentation, drying, and ripening. Common fermented sausages in the Irish market are salami, pepperoni, and chorizo, which are derived from traditional processes from other European countries. Salamis are known to be fermented meat products from Italy and Germany (i.e., *rohwusrt*), while chorizo is from Spain. Raw or uncooked meat products have not been treated with the required temperature for pasteurization, which increases the risk of pathogen growth. However, various processes such as cold smoking, drying, fermentation, direct acidification, and curing can be used to add additional barriers to microbial spoilage and the growth of harmful microorganisms. These processes also result in a variety of meat products that have distinct characteristics. 

Preserving and enhancing cured meat products, whether whole meat cuts or comminuted, often involves a combination of fermentation and drying processes. Fermentation is carried out traditionally using the indigenous microorganisms present in the meat. Nowadays, predetermined microbial starters are added to achieve the desired functionality and organoleptic attributes. Bacteria belonging to the genera *Lactobacillus*, *Pediococcus*, *Staphylococcus*, and *Streptococcus* are commonly used [47]. Certain bacteria such as *Lactobacillus sakei*, *Lactobacillus plantarum*, *Leucononstoc strains*, *Staphylococcus carnosus*, and *Staphylococcus xylosus* are also incorporated to allow the conversion of nitrates into nitrites in meat curing reactions as they express nitrate reductases at anaerobic conditions [23]. Commercially available freeze-dried starter cultures such as CHR Hansen Bactoferm ^®^ are used on these types of products. These microorganisms produce lactic acid from the metabolism of sugars at appropriate conditions, thus reducing the pH of the product and inhibiting the growth of pathogenic bacteria [33]. This reduced pH also facilitates the color fixation function of nitrites. After the fermentation phase of the processing, these meat products are subsequently dried and ripened before consumption. This is a long process that takes advantage of the preservative action and flavor development of fermentative microorganisms, and the moisture removal by the drying process. 

Dry-fermented whole pieces of meat are typically dry-cured by rubbing curing salt, which contains sodium chloride, and curing agents onto the meat until fully covered. It is then kept at a refrigerated temperature of approximately 5 °C for 2–3 weeks. Spices such as pepper, coriander, mustard, and juniper berries may also be added for flavor, depending on the variant being produced. Products found in the Irish retail markets are also of foreign origin, particularly from Spain (such as Serrano ham) or Italy (including prosciutto, coppa, and Bresaola), and typically comply with specific specifications to claim their name and origin. Parma ham is a dry-aged meat product that originates from Parma town in northern Italy. This meat product does not use nitrite for curing but forms and maintains red color in the meat during storage [48]. This is of particular interest to meat product developers when developing nitrite-free meat products with similar characteristics in terms of red color stability similar to those that are cured with nitrites. 

There are no documented indigenous, fermented sausage-type meat products in Ireland. The term for locally manufactured sausage is the Irish breakfast sausage; however, the process of production does not involve fermentation nor drying. This product is made from pigs slaughtered and processed in Ireland. Lean meat and fat are minced and mixed together with salt, mild spices, and seasonings for flavor and stuffed into natural casings, and is sold raw. The ingredients are similar to those of emulsified cooked sausages (i.e., they contain additives such as phosphates as a stabilizer and ascorbate as an antioxidant), except that it does not contain nitrites as preservatives; rather, sodium metabisulfite is used. 

### 2.3. Safety Aspects and Regulation of Cured Meat

Nitrate is naturally occurring in human saliva and is actively secreted by the salivary glands, or may also be produced by oral microflora [29]. The enzyme nitrate reductase from this natural microflora is also capable of converting nitrate to nitrite, which may also be transformed into nitric oxide during oral digestion in the mouth and the stomach. According to the recent risk assessment by the European Food Safety Authority (EFSA), the acceptable dietary intake (ADI) of nitrites among all age groups will be exceeded if exposed to all possible dietary sources (e.g., natural presence, food additives, and process contamination) [8]. The issue with nitrites, nitrates, and their intermediates in curing reactions, such as nitrous acid and nitric oxide, is that, being nitrosating agents, they can form various N-nitroso compounds (NOCs) such as N-nitrosamines, which have gathered public health concern [49]. A study by the IARC [7] highlighted a possible linkage between red and processed meat and colorectal cancer. An EFSA Panel on Contaminants in the Food Chain stated that ‘meat and meat products’ are the main food category that contributes to nitrosamine exposure, among the five food categories analyzed [8]. The National Cancer Registry Ireland (NCRI) has listed processed meat as one of the risk factors of cancer [50]. N-nitrosamines are produced when nitrosating agents such as nitrites react with secondary amines derived from protein and lipid oxidation products in meat. Nitrosamines can also be produced endogenously upon consumption of meat products that contain nitrosating agents. Common N-nitrosamines that consumers can be exposed to from consuming cured meat products include N-nitrosodimethylamine (NDMA), N-nitrosodiethylamine (NDEA), N-nitrosopiperidine (NPIP), and N-nitrosopyrrolidine (NPYR) [7]. These compounds fall under groups 2A or 2B, which means that they have a potential carcinogenic effect [7,50]. The types and amounts of nitrosamines in processed meat products vary depending on the matrix and processes involved. The formation of nitrosamines in meat products is a complex process influenced by various factors, including microorganisms that can convert nitrates to nitrites and degrade proteins to amino acids and amines. Nitrosamine can also develop in meat during the production process, home cooking, and in the digestive tract after ingestion [51]. High nitrite consumption can also affect iodine metabolism and reduce iodine absorption by the thyroid [52].

The level of incorporation of nitrates and nitrites by meat processors in Ireland is based on the limits set by Commission Regulation (EU) 1333/2008 on specific meat products. The FSAI regulates cured meat products to ensure compliance with standards of identity and food safety. The use of salts, salts of nitrates, and nitrites together with other additives (i.e., phosphates and ascorbates) on meat must comply with European Commission [19] Regulation 1333/2008/EC, as amended on food additives, which has been incorporated into Irish legislation by S.I. No. 330 of 2015. Cured meat products are classified as non-derogated and derogated. Non-derogated products include meat products cured by injection or dry curing for less than four days, sterilized meat products, cure-in-bag meat products that are injected with curing solution and not immersed, and uncooked cured tongue. It is important to note that sodium and potassium nitrate are only permitted in non-heat-treated meat products, while nitrate may be present in some heat-treated products due to natural conversion. Derogated meat products are produced through traditional curing processes and have specific derogations provided for in Directive 2006/52/EC [53]. The maximum limits for nitrates and nitrites in derogated products considered to be particular EU Member State national products relate to the maximum residual levels permitted in the finished product at the end of the production process. The amount of additives in cured meat products is controlled based on the in-going amount (i.e., in the formulation, prepared brine, and the injection/curing immersion uptakes). Based on the outgoing legislation on food additives, the maximum in-going amount of each nitrate and nitrite (reported as the sodium salt form) on non-heated meat products is 150 mg/kg. However, recent changes in the EU regulation allows meat processors to apply this existing limits only until October of 2025, as the limits of the addition of nitrites on meat products during manufacturing is reduced. From October 2025, the maximum limit for the addition of nitrate ion on non-heated meat products is set to 90 mg/kg, while it is 80 mg/kg of input nitrite for both non-heated and heat-treated meat products [9]. This nitrite limit is also aligned with the Codex General Standard for Food Additives for heat-treated processed meat, poultry, and game products in whole pieces or cuts, and with processed comminuted meat, poultry, and game products [54]. The same 80 mg/kg nitrite ion limit (equivalent to 120 mg/kg sodium nitrite) is also set as the maximum allowable level of incorporation for pumped and massaged bacon, and for immersion cured bacon in the USA [55]. Denmark has a more stringent maximum allowable level of incorporation of sodium nitrite on meat products than the rest of the EU. The Danish national regulation sets a maximum of 60 mg/kg of sodium nitrite as the permitted level for meat products [56]. In the recent EU regulation, the residual amount of nitrite and nitrate ions of non-heat-treated and non-sterilized heat-treated meat products is also set to 45 mg/kg NO_2_^−^ and 90 mg/kg NO_3_^−^, respectively, throughout the shelf-life of the product. In a study conducted by Crowe et al. [57] on bacon products in the UK, it was shown that the mean residual nitrite concentration for all bacon samples is 10.80 mg/kg, which is significantly lower than the allowable limit aligned to the recent change in EU legislation on food additives. Similarly, in Italy, the levels of residual nitrite on cured meat products (0.76–25.67 mg/kg) analyzed were below the allowable limit for finished products, though large variations were observed across various meat products. On the other hand, the levels of residual nitrate (up to 188 mg/kg) are particularly high on most of the meat products analyzed [58]. In Iran, the mean residual nitrite of the meat products analyzed is 55.18 ppm (equivalent to the same value in mg/kg), which is below their national allowable limit of 80 mg/kg nitrite [59]. While there are other possible natural dietary sources of nitrates and nitrites, such as in the natural interconversions in digestion, and from vegetables and root crops, reducing or removing them as food additives on cured meat products are beneficial strategies to reduce the exposure of consumers to these nitrosating agents and thus to N-nitrosamines. 

## 3. Opportunities in the Reformulation and Processing of Cured Meat Products

Processing treatments are applied to meat in order to produce the necessary quality and safety parameters of products. However, consumers are becoming more mindful of the products they eat, and the product image of processed meats is negatively affected as these are perceived as unhealthy [12]. There is clearly an increased demand for meat products that have ‘clean labels’. Though it is argued that there should be clarity on the meaning of ‘clean label’, it generally implies that it should be free from chemical additives and preservatives [60]. This prompts food product developers to design products in such a way as to suit the demands of consumers without compromising quality and safety. Furthermore, efforts from regulatory agencies and the governments focus on initiatives of improving the safety and nutritional profile of meat products [61]. This is challenging because certain food technologies were introduced to favor certain desirable aspects. Chemical additives and preservatives are utilized to improve the quality and shelf-life of food. However, problems of this type arise when unintended undesirable compounds are produced during processing and storage. The development of meat products that would meet these demands involves the correct balance when considering nutrition, safety, and consumer acceptance. In developing consumer-packaged food products, the following aspects are important considerations: (1) formulation, (2) processing technologies, and (3) storage and packaging technologies [62]. These are interrelated and greatly affect each other, so it is important to consider each of these during the course of the development process.

### 3.1. Reformulation of Cured Meat Products

The formulation of a food product is related to ‘what is inside the product’. Food is a matrix of biochemical molecules such as water, protein, fat, carbohydrates, minerals, and vitamins; certain other chemicals are introduced in order to obtain the desired quality. When talking about formulation, it involves the intended quantity of each of these components, either added or mixed. Based on the common meat products consumed in Ireland, salt, saturated fat and chemical additives such as phosphates, and preservatives (e.g., nitrites and nitrates) are of concern to consumers as they become more aware of the effects of these ingredients on their health [12].

As mentioned, meat curing involves the use of salt and nitrites and also encompasses complex chemical and biochemical reactions in the meat matrix that provide the desirable characteristics [20]. Meat product developers face this challenge to look for alternative ingredients, or to apply innovative food processing techniques in order to provide the same functions of nitrites as cost effectively as possible [20,63]. However, many researchers agree that it is challenging to have a single ingredient for a small dosage use that can have the same wide range of technological functions as nitrites [64,65]. Several research studies have been conducted to look for ‘cleaner’ and more natural ingredient and/or processing techniques as alternatives to the application of synthetic nitrates and nitrites on cured meat products. Extensive reviews have also been published to consolidate these research studies, presenting the different approaches to provide knowledge on alternatively cured meat products [25,29,64,66]. Based on existing studies, the following strategies have been used:

- Natural sources of nitrates and nitrites from plant-based sources, or products of microbial fermentation;
- Extracts from plants, fungi, and dairy sources that can provide the technological functions of nitrates and nitrites (e.g., as colorant, antioxidant, antimicrobial) on meat products;
- Emerging processing technologies;
- Packaging technologies;
- Combination of interventions/hurdle technology.

Natural sources of nitrates and nitrites, such as broccoli, celery, spinach, lettuce extracts [67,68,69,70], parsley [71], red beet extract and Swiss chard extract [72], and radish [73,74], for meat application were explored for a ‘clean label’. The use of these plant-based extracts involves fermentation to convert the naturally occurring nitrates into nitrites, which is the more active compound for color fixation, control of microbial growth, and cured flavor development [66]. This conversion needs the correct conditions in order to proceed with the desirable reactions. Fermented celery extract with pre-converted nitrites is popularly used as a natural source of nitrite for meat product applications in the United States market [75]. However, this poses challenges in the European market, where celery is considered as an allergen [76]. Table 3 shows some of the plant extracts used in several studies as nitrite alternatives for different meat product applications.

Many of the nitrite alternative ingredients were extracted from plant sources such as raspberry [60], tea polyphenols [85,99], red grape pomace [100], and black currant leaf extract [80]. Other sources of natural colorants, such as carmine from cochineal insects [101] and microbial pigment from yeast *Monascus purpureus* [79], were also explored. These extracts individually provide the desired color or antioxidant properties in the meat products. Thus, combinations of these extracts were necessary to provide the same quality and stability as the ones treated with synthetic nitrites. In some studies, innovative treatment methods were used for the extraction of these alternative ingredients. For example, plasma treatment technology was applied to winter mushrooms in the development of nitrite-reduced canned ground ham [95]. Similar technology was also applied to milk powder as an ingredient to replace nitrites in pork sausage [102]. However, the aroma profile of the finished product was negatively affected. Freeze drying was used in the pre-processing of shiitake stipes to be used on fermented sausages to improve the stability towards the lipid oxidation and growth of pathogenic microorganisms [103]. The limitation of these alternative ingredients is that they do not provide all the functions of nitrites in meat products [104]. In addition, the extraction and purification of these alternative ingredients require optimization studies regarding their applications on different meat products. 

Rocchetti et al. [105] used hurdle technology by applying low-temperature treatment to the ripening and drying processes of nitrate/nitrite-free fermented sausage, which provided comparable microbiological and metabolomics profiles to the samples with nitrites. 

### 3.2. Emerging Food Processing Technologies and Innovative Technologies for Cured Meat Products

Aside from the incorporation of alternative ingredients to meat products, there are several new processing techniques being applied to produce diverse ranges of cured meat products. These technologies offer different mechanisms of preservation that can complement the reduced use or total removal of nitrates and nitrites in the formulation. Table 4 shows some of the emerging processing technologies that were explored to meat curing. 

Some of the emerging technologies that were applied to the curing of meat products are plasma treatment, high-pressure processing, pulsed electric field, and ultrasound treatment. The use of atmospheric, non-thermal plasma treatment was applied to cured roasted lamb [106]. The results show that the sensory quality attributes were improved with a 30 min application of plasma versus the use of the same product with sodium nitrite, but it recommended examining the microbial stability of the products that have undergone this processing. An alternative nitrite source for cured meat product applications was produced by using plasma technology on plant protein preparation solutions [111] and water [112]. Both suggested that these treated materials be applied to cured meat products. Plasma treatment uses partially ionized gas as a treatment in water in order to generate reactive oxygen and nitrogen species such as peroxynitrites, nitrate, and nitrite, which are essential in the curing process [113]. High-pressure processing (HPP) is a novel processing technology that has been used by several researchers on meat applications. It takes advantage of the detrimental effect of extremely high levels of pressure in order to destroy microbial cells without the use of heat. Inguglia et al. [11] and Rodrigues et al. [114] highlighted the potential of this technology for the reduction of salt in meat products. They recognize that there are several challenges that need to be addressed in order to improve the flavor perception and safety of sodium-reduced products. This can be achieved by combinations of novel processing treatments such as HPP. Yang et al. [115] affirmed the possibility of using this technology to reduce both fat and salt on pork sausages. HPP is usually used in combination with other treatments when the intention is to reduce or remove the amount of nitrites. Myers et al. [88] used HPP in combination with commercial vegetable juice powder, a natural source of nitrite, for cooked ham. The use of HPP (600 MPa for 8 min) enhanced the microbial stability of dry cured meat against *Listeria monocytogenes* [116]. This technology was also explored in relation to reducing phosphate in meat product formulations. However, the results of O’Flynn et al. [117] suggest that an elevated level of pressure of 300 MPa negatively affected the texture of low-fat sausage. The application of 150 MPa has potential for reducing phosphate levels in sausages without significant changes in their functionality and improved acceptability. These results show the challenge of favoring both the quality and safety attributes of the product while introducing new technologies. Thus, appropriate optimization of the process parameters to the desired quality and safety parameters are important. Ultrasound technology has also been explored for its application in meat processing, which would hasten the curing process in meat [118,119,120]. The results of the study by Tong et al. [110] reveal that this technology can hasten curing in chicken breast meat and improve the tenderness and water-holding capacity, and can even retain flavor that could have been lost due to the application of phosphates. This would have potential in reducing the use of phosphates on meat products. Similarly, the pulsed electric field (PEF) has been explored for its application on meat and meat products. According reviews by Bhat et al. [121] and Gómez et al. [122], PEF is postulated to cause the cell membranes on the meat to release minerals such as calcium and enzymes that can provide both desirable and undesirable effects on the matrix, depending on the application. Tomasevic et al. [123] highlighted some of the grey areas regarding the techno-functional effects of PEF on meat quality, such as cooking loss and color. This technology is relatively new for cured meat applications, and it is important to consider the different aspects of sustainability (i.e., economic, environmental). 

Aside from the new processing and preservation technologies being explored regarding cured meat product applications, one product of interest when it comes to nitrite removal is understanding the mechanisms behind the color stability of Parma ham. This traditional meat product from northern Italy does not use nitrites during curing, but forms and maintains the red color during storage [48,124]. Researchers elucidated that this quality is attributed to the formation of zinc protoporphyrin IX (ZnPP), a red pigment produced by bacteria in dry-fermented meat [125]. However, the mechanism of the formation and stability of this desirable color is not yet fully understood. Another possible approach on producing the desired color of cured meat without using any additives is understanding the role of L-arginine on the production of nitric oxide (NO) through the nitric oxide synthase (NOS) system [126]. In a consumer study conducted by Chambers et al. [127], this mechanism is referred to as an amino acid-based alternative meat curing system. The supplementation of arginine was applied to the diet of steers, and the results show that diets supplemented with arginine and a combination of arginine and lysine gave a superior color stability for beef loins [128]. Zając, Zając, and Dyvas [129] investigated the effects of the direct addition of nitric oxide synthase and arginine to fermented sausages. However, their results showed that the products that contain the added NOS enzyme and arginine did not have the same color as their nitrite-cured counterparts. This leads to a further recommendation to optimize the pH, time, and temperature in handling the NOS enzyme. Several fermentative microorganisms have been studied that exhibited promising nitric oxide synthase activity that can contribute to the formation of the cured meat pigment. Among the three tested species of coagulase negative staphylococcus that can produce nitric oxide through NOS enzyme catalyzed reaction, *Staphylococcus vitulinus* resulted in a higher nitric oxide–myoglobin formation than *Staphylococcus carnosus* and *Staphylococcus equorum* [130]. In addition, *Lactobacillus fermentum* AS1.1880 is also confirmed to have good NOS activity that leads to the production of nitrosomyoglobin on cured meat [131]. Understanding the formation of the Zn-porphyrin pigment and the mechanism of arginine–nitric oxide reactions can be useful for the development of nitrite-free meat products. 

Packaging technology is also an important aspect in this product development. Chatkitanan and Harnkarnsujarit [132] incorporated nitrite as part of the active packaging system for raw pork. This contributed to microbial stability and the red color of the product. Similarly, nitrite-embedded commercially available film was also tested as packaging for fresh beef, and it was seen to retain the bright red color in the display storage [133]. Another active packaging technology intended for cured sausages was introduced by Hamann et al. [134]. They incorporated green tea extract and gelatine onto edible film, which further contributed to stability against lipid oxidation.

As nitrates and nitrites are already sought to be reduced or removed on meat products, there is a risk that the desirable functionalities (i.e., color fixation, flavor development, and preservation against spoilage and pathogens) in the food matrix will also be lost. Meat processors must consider other ways to compensate these functions without sacrificing quality, safety, and consumer demands on this meat product category. The mentioned emerging technologies (i.e., plasma treatment, high-pressure treatment, ultrasound) are relatively new technologies when it comes to cured meat product application, as they offer different mechanisms of preservation and quality improvement. However, one of the main drawbacks in the use of these new technologies is that they usually require large investments in terms of capital outlays and further research to ensure the necessary product performance in the market. It is then crucial to consider the impacts of these technologies on the overall quality and safety of the food being produced, also the benefits to businesses and to the environment.

### 3.3. Understanding Consumers’ Preference Considerations: A Demand Driven Approach

Consumer insights are important for the development of cured meat products. Many of the studies on the reformulation and application of innovative processing technologies on cured meat products involved consumer acceptability studies alongside testing the physico-chemical and microbiological quality parameters [60,77,103]. Consumers are at the final stage in the production and consumption chain, and it is useful to identify which factors affect their behavioral patterns [135]. Consumers’ preferences, behavior, and perception of meat and meat products are complex and do not depend only on the appearance and sensory properties of the meat but also on psychological and marketing aspects [136]. There are concerns about adding ingredients that are perceived as healthy to meat products due to opposition to the idea that the product might be overly processed, low familiarity with processed meat as a functional food, and uncertainties regarding the overall health characteristics of the final product [137]. Studies have shown that consumers have a higher intention to purchase meat products that contain natural additives (e.g., herbs and berries) rather than chemical ones [138]. This preference is due to the perceived harmfulness of chemical additives. In addition, consumers consider the taste, appearance, price, and information level of meat products with these natural ingredients [139]. In a study among Irish consumers of processed meat, it was shown that their purchase intention was primarily influenced by the price and the type of meat used in the product, and this was followed by the presence of ingredients that were perceived as being healthy and by salt and fat contents, and hams and sausages are preferred for formulation over beef burgers [140]. Similarly, a study on the incorporation of herbs and berries for the preservation of meat products also indicated that price is an important main determinant of consumers’ purchase intention, with information of the incorporation being clear to them [138]. Most likely, products reformulated to be healthy (i.e., reduced fat, salt, and preservatives) results in a higher price than the original product, which might hinder acceptance for low-income consumers [141]. The extent that price influences purchase decisions is also affected by other values that the product would provide (e.g., nutritional value, sensory attributes, relative price with similar products), along with the socio-demographic profile of consumers (e.g., income, lifestyle, etc.) [140]. 

## 4. Challenges and Future Prospects

Due to the association of cured meat products with public health concerns regarding nitrosamines, the presence of chemical additives, and high levels of salt and fat, reformulation seems to be one of the possible directions in improving the nutritional properties of these products. Multiple studies have focused on the reduction and/or removal of essential ingredients in curing; the ingredients sought to be reduced or removed are salts of sodium (e.g., sodium chloride, sodium polyphosphates, sodium nitrate, and sodium nitrite) [10,142]. These all align with the WHO’s recommendation to reduce the intake of sodium, in which excessive consumption is linked to health problems such as cardiovascular diseases [143]. Plant-based ingredients are presently the leading solutions for these products, as consumers associate them with being healthy [144]. In terms of the identity of cured meat products, the removal of curing agents on these products without a mitigating intervention in their manufacture would challenge the uniqueness of this meat category. Different variants of ham are recognized as meat products that undergo a curing process, where nitrites are used as one of the essential ingredients [145]. However, the removal of these additives would raise the question of the definition of curing, and this may have implications on the categorizing and labelling of products with alternative ingredients and/or those that would undergo an alternative process. For example, in the USA the removal of synthetic nitrates and nitrites (i.e., sodium and potassium salts of these ions) and the replacement of natural sources such as celery extracts has allowed manufacturers to use ‘uncured’ and ‘no nitrates/nitrites’ on the package labels, according to the existing regulations [146]. However, consumer groups have raised the question of whether this labelling might mislead consumers, as the same nitrate/nitrite compounds are still introduced, and may provide the same concern as the formation of nitrosamines [147]. In Ireland, the FSAI made it clear that the use of alternative ingredient (i.e., celery extracts and other ingredients that are used for the intended technological purpose similar to that of nitrites) is considered as the deliberate use of a food additive and should comply with food additive legislation [148]. Innovative processing solutions such as high-pressure processing, ultrasound, and plasma treatments are also being explored regarding the development of the cured meat category [88,108,119,149]. However, economic sustainability is one of the challenges in the implementation of these proposed interventions [123]. It is known that nitrites are relatively cheap ingredients, wherein a very low dosage is required to offer a wide range of technological functions, as compared to the application of plant extracts and innovative processing, where they usually require a high cost of investment [63]. Sustainability in the industrial scale application is also another crucial consideration. The EU has revised the allowable input of nitrites on meat products, which is from 150 mg/kg of sodium nitrite to 80 mg/kg of nitrite ion (equivalent to 120 mg/kg of sodium nitrite salt) for meat products [9]. There are still current challenges regarding balancing the desirable effects of nitrates and nitrites on the quality and safety of meat products, as well as on the undesirable effects of these chemical additives on consumers’ health. 

## 5. Conclusions

There is a variety of meat products that are known for their exceptional quality in the Irish market. For many years, they have been an important part of the Irish diet. However, the cured meat category has been gaining attention due to the presence of nitrites, which are associated with certain health concerns. The recent changes in EU legislation on the reduction of nitrate and nitrite limits is timely, as meat products are gearing towards ‘clean labels’, and because consumers are becoming more aware of the importance of healthy diets. However, these curing agents were proved to be essential in the quality and safety of these products in terms of providing the desired color, flavor, and stability against spoilage and pathogenic microorganisms. The reformulation of traditional meat products might possibly challenge their distinct intrinsic characteristics when these curing agents are reduced or even replaced by alternatives. There have been significant developments in exploring various alternative ingredients, as well as the use of novel processing and packaging technologies, to fulfil the functionalities of the aforementioned compounds on meat products. Nevertheless, there are still many aspects that need to be addressed, especially the applicability on a wide variety of meat products and sustainability at the industrial scale.

## Figures and Tables

**Table 1:** Common whole meat cuts used for the production of cured meat products [17].

Type of Meat	Whole Meat Cut	Description	Uses and Applications
Pork	Ham leg	Hind leg of the animal that is composed of the silverside and topside muscles	Ham, gammon
	Loin	Wide, thick, and rectangular cut from the midsection of the back portion that runs from the shoulder to the rear	Back bacon
	Side	Located directly beneath the loin; belly part; contains no bones	Streaky bacon, pork belly
	Butt	Located at the thicker portion of the pork shoulder and includes parts of the neck	Buckboard bacon
	Picnic shoulder	Located at upper portion of the foreleg and below the butt	Shoulder ham
Beef and veal	Brisket	Tough and course textured muscle; contains a substantial percentage of fat	Corned beef brisket; dry cured pastrami

**Table 2:** Basic listed ingredients of different selected commercial Irish meat products.

Meat Products	Basic Ingredients	Variants
Bacon	Pork, water, salt, antioxidant (sodium ascorbate), preservatives (potassium/sodium nitrate, sodium nitrite)	Traditional/Irish
		Cuts: back bacon rashers, streaky, bacon chops, lardon, joint, medallion, eye loin
		Flavor: traditional cured, smoked, smoked paprika and orange blossom, smoked dry cured whiskey, with Bourbon, maple syrup and cinnamon glazed, orange blossom and honey and ginger
Cooked ham (deli-style)	Pork, water, salt, stabilizer (one or the combination of the following: salt of diphosphate, triphosphate, polyphosphate), antioxidant (sodium ascorbate), preservative (sodium nitrite and sodium/potassium nitrate)	Traditional/Irish cure
		Size and form: hand cut, carved, large slice, shaved, wafer thin, deli style, center cut
		Flavor: smoked, roasted, oven baked, slow cooked, chipotle, barbecue, 7-day dry aged
		Glazing: firecracker, sweet, honey roast, orange and wildflower, honey
		Coating crumbs: breadcrumbs, ciabatta
Cooked emulsified meat	Pork, salt, spices, flavorings, preservative (sodium nitrite),	Frankfurter, luncheon roll, Mortadella, Danish ham Format: deli-style cooked slices, canned
Cured beef products	Beef, water, salt, preservative (sodium nitrite) Cure-in-bag corned beef and spiced beef also contains dried glucose syrup and antioxidant (sodium ascorbate)	Irish corned beef Irish spiced beef Format: whole meat piece (cure-in-bag), deli-style cooked slices, canned
Cured offal (ox tongue)	Ox tongue, salt, sugar, stabilizer (one or a combination of the following: salt of diphosphate, triphosphate, polyphosphate), beef gelatine, antioxidant (sodium ascorbate), preservative (sodium nitrite)	Format: deli-style cooked slices, canned
Dry-fermented/salted meat	Pork (except for Bresaola), salt. Preservatives (sodium nitrate, sodium nitrite)	Prosciutto (crudo, Parma (does not contain preservatives) Bresaola (made from beef) Coppa Serrano Paleta de Cebo (made from pork shoulder)
Fermented sausages	Pork, salt, preservatives (sodium/potassium nitrate, sodium nitrite), antioxidant (sodium ascorbate), dextrose, spices and flavorings	Salami (variants: Milano, Ventricina, Napoli, Toscano, German-style) Pepperoni (variants: regular, hot, mild) Chorizo (variants: regular, spicy)
Gammon steak/Ham fillet	Pork, water, salt, antioxidant (sodium ascorbate), preservatives (sodium nitrite and sodium/potassium nitrate)	Traditional/Irish cure
		Size: Gammon steak, 1 kg, 1.5 kg, 2 kg
		Flavor: Smoked, unsmoked
Pudding	Cereal grains (one or a combination of the following: oatmeal, pearl barley, oatflakes), wheat flour, bread crumbs, blood (for black pudding, one or a combination of the following: pig blood, beef blood), animal fat (one or a combination of the following: pork fat, beef fat), pork rind, cured meat (contains pork, water, salt, sodium phosphates, sodium ascorbate, sodium nitrate, sodium nitrite), onion, spices, and seasonings	Black pudding—contains blood White pudding—does not contain blood
		Regular, gluten-free
		Form: Regular, ring

**Table 3:** Plant extracts applied to meat products to provide some functions of nitrite functionality, totally or partially, based on the most recent studies.

Plant Extracts	Product Application	Processing Applied to Meat	References
Arugula and barberry extract	Heat-treated fermented sausages *Pediococcus* *acidilactici*, *Lactobacillus Plantarum*, and *Staphylococcus carnosus*	Mincing, fermentation, cooking	[77]
Banana inflorescences extract of male flowers, as antioxidant	Sausage (uncooked)	Mincing	[78]
Beetroot solution and *Monascus* color with *Lactobacillus fermentum* RC4 and *Lactobacillus plantarum* B6 as starters	Cured meat (unsmoked Chinese bacon)	Dry fermentation	[79]
Black currant leaf extract	Canned pork (50 ppm NO_2_^−^)	Mincing and cooking	[80]
Chia products (seeds, flour, and a coproduct from cold-press oil extraction)	Frankfurters	Mincing and cooking	[81]
Chitosan and radish powder	Fermented cooked sausages	Mincing, fermentation, cooking	[82]
Concentrated parsnip fermented juice with 6237.5 ppm nitrite content Hawthorn extract	Pork mince	Mincing	[83]
Coriander essential oil	Cooked pork sausages	Mincing and cooking	[84]
ε-polylysine nanoparticles (ε-PLNs) combined with plant extracts (including green tea, olive leaves, and stinging-nettle extracts)	Frankfurter-type sausages (minced and cooked)	Mincing and cooking	[85]
Freeze-dried celery	Cold-smoked sausages	Mincing, dry fermentation	[68]
Grape seed extract and chestnut extract	Italian Cinta Senese sausages dry-fermented	Mincing, dry fermentation	[86]
Green tea extract, rosemary extract, as antioxidant	Bologna type sausages (cooked)	Mincing and cooking	[87]
Vegetable juice powder and high-pressure processing	Cooked ham	Mincing and cooking	[88]
Grape seed extract and olive pomace hydroxytyrosol; Chestnut extract and olive pomace hydroxytyrosol	Cinta Senese dry-fermented sausages	Mincing, dry fermentation	[89]
Japanese radish derivatives with starter culture containing *Staphylococcus carnosus* ssp. and *Staphylococcus carnosus.*	Restructured cooked hams	Mincing and cooking	[73]
*Kitaibelia vitifolia* extract	Fermented dry sausage in natural pork casing	Mincing, dry fermentation	[90]
Mustard seed and acid whey	Cooked sausage	Mincing and cooking	[91]
Natural nitrite from fermented hydrated spinach powder	Cooked meat (4 days cured at chilled, cooked)	Mincing and cooking	[92]
Natural pre-converted nitrite sources from fermented spinach, lettuce, celery, and red beet using *S. carnosus*	Raw and cooked pork sausage	Mincing and cooking	[70]
Oregano:olive vegetation water = 1:7 (*w*/*w*) green tea:blueberry:water = 0.5:0.5:7 (*w*/*w*/*w*)	Pork salami (with *Lactobacillus sakei* and *Lactobacillus curvatus* (Salum 20), *Staphylococcus xylosus*, and *Staphylococcus carnosus* (Salum 30)	Mincing, dry fermentation	[93]
Parsley extract and starter culture	Mortadella-type sausages (cooked sausage)	Mincing and cooking	[71]
Grapeseed, green tea and olive, and addition ascorbic acid	Salami	Mincing, dry fermentation	[94]
Plasma-treated winter mushroom powder	Canned ground ham	Mincing and cooking	[95]
Radish and beetroot powders	Fermented dry sausages	Mincing, dry fermentation	[74]
Raspberry water extracts	Pastırma (dry cured)	Dry curing	[60]
Red beet extract and Swiss chard juice extract	Emulsion-type sausages (with *Staphylococcus carnosus* (Bactoferm CS-300^®^)	Mincing, fermentation, cooking	[72]
Rosemary (*Rosmarinus officinalis*) extract, celery (*Apium graveolis*)	Colonial type salami	Mincing, dry fermentation	[96]
Sorghum red pigment powder	Canned meat	Mincing and cooking	[97]
Spinach emulsion	Meat batter, cooked sausage, roasted sausage	Mincing and cooking	[98]
Theaflavins, tea polyphenols, Vitamin C	Cured sausage	Mincing and cooking	[99]

**Table 4:** Application of some emerging food processing technologies to meat products.

Emerging Processing Technology	Product Application	Processing Applied to Meat	References
Atmospheric non-thermal plasma treatment	Roasted lamb and beef	Roasting	[106,107]
Cold atmospheric plasma	Roasted beef (4 cm × 4 cm × 2.5 cm)	Roasting	[107]
High-pressure processing in combination with vegetable juice powder	Cooked ham	Mincing and cooking	[88]
High-pressure processing	Fermented sausage	Mincing, fermentation, and drying	[108]
Plasma-activated milk powder	Pork sausages	Mincing and cooking	[102]
Pulsed electric field	Dry-fermented sausage	Mincing, fermentation, and drying	[109]
Ultrasound-assisted curing of meat	Raw chicken breast (4 cm × 4 cm × 3 cm)	Raw	[110]

## Data Availability

The original contributions presented in the study are included in the article, further inquiries can be directed to the corresponding author.
